# Regulating steric hindrance in difunctionalized porous aromatic frameworks for the selective separation of Pb(II)

**DOI:** 10.1016/j.isci.2023.108274

**Published:** 2023-10-19

**Authors:** Xuan Ding, Jiayi Liu, Hui Shi, Zhou Yi, Lei Zhou, Wei Ren, Penghui Shao, Liming Yang, Derun Zhao, Yun Wei, Xubiao Luo

**Affiliations:** 1 Key Laboratory of Jiangxi Province for Persistent Pollutants Control and Resources Recycle, Nanchang Hangkong University, Nanchang 330063, P.R. China; 2 School of Life Science, Jinggangshan University, Ji’an 343009, P.R. China; 3 School of Computational Science and Electronics, Hunan Institute of Engineering, Xiangtan 411104, P.R. China

**Keywords:** Computational physics, Membranes

## Abstract

Efficient and selective removal of Pb(II) from wastewater with complex matrix remains a challenging task. Porous aromatic frameworks (PAFs) with predesigned functional building blocks provide a favorable platform for the selective separation of Pb(II). Herein, the bifunctional SPAFs with the introduction of -OH and -SO_3_H were synthesized through rationally optimizing their steric hindrance. As a result, the SPAF-0.75 exhibits favorable adsorption capacity of Pb(II) (212.34 mg g^−1^), which is 22 times larger than pristine framework. Competition experiment indicates that SPAF-0.75 possess the selective removal of Pb(II) without interfering from co-existing metal ions. The removal rate of SPAF-0.75 still retain at 100% after six successive cycles. The DFT calculation illustrates that -OH and -SO_3_H are co-participate in the process of capturing Pb(II), revealing SPAF-0.75 preferred removal of Pb(II) owing to the lowest adsorption energy (Δ*E*_*ab*_ = −3.99 eV). This study extend the understanding of the structure-property relationship and facilitate new possibilities for PAFs.

## Highlights

• Bifunctional SPAFs was prepared for removal of Pb(II) from wastewater
• The modulate SPAF-0.75 features the most suitable steric hindrance of active sites
• Selective separation of Pb(II) was achieved by the optimum synergistic effect
• The novel idea extend the perceive of structure-property relationship of adsorbents

## Graphical abstract

**Figure:**
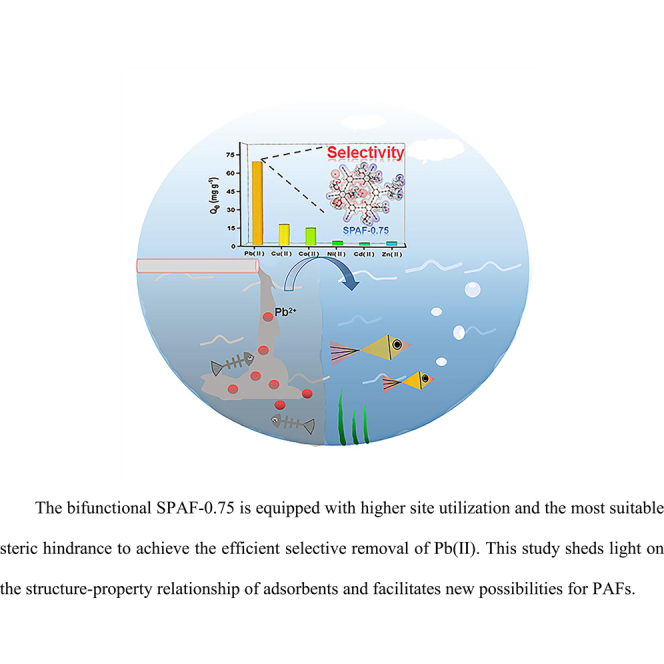

## Introduction

Heavy metal ions are derived from electronic, battery, and electroplating industry have attracted extensive attention account for the persistently contamination to water.^1^ Pb(II) is common pollutant that usually released, which cannot be degraded and may accumulate in living organisms through food chain to pose hazardous damage for ecosystem and human being.^2^ In view of this, it is necessary to take some effective measures to control Pb(II) pollution in water. Among the numerous water treatment technologies, adsorption receives a lot of attention and is recognized as one of the most attractive and applicable technologies due to its eco-friendly advantages, convenient operation, and generation of non-toxic by-products.^3^^,^^4^ The key to the adsorption method is the selection of adsorbents. To date, tremendous effort has been made to establish numerous materials with diverse techniques including limonitic laterite, magnetic biochar, functionalized chitosan beads and MOFs to treat Pb(II) with low limit of detection (LOD) and large adsorption capacity.^5^^,^^6^^,^^7^^,^^8^ Nevertheless, the existing adsorbents are still inevitably interfered by competing heavy metal ions, especially Cd(II) and Cu(II) under the process of removing Pb(II) from wastewater. It is imperative to develop innovative materials and novel strategies for conquer the dilemma in efficient selective removal of Pb(II).

Porous Aromatic Frameworks (PAFs), on account of their intriguing merits of abundance, sustainability, adjustable structures and easy modification, the PAFs as favorable adsorbents in heavy metal ions removal had been attracted wide attention.^9^ In previous studies, to cope with the treatment of Pb(II) in effluent, hydroxyl groups were generally used to prepare adsorbents with special functions.^10^^,^^11^ The practical wastewater environment generally presents the coexistence of multiple heavy metal ions, previous field investigations of researchers (including the antimony mine area, lead-zinc coal mine area, and so forth Hunan, China) demonstrated that the wastewater around the mining area mostly existed in the coexistence of multiple potentially toxic elements (PTEs) dominated by Cd(II), Pb(II), Cu(II), Zn(II), Co(II)).^12^ However, these adsorbents lack the ability to selectively capture of Pb(II) in wastewater. Sulfonic group exhibit excellent chelating properties for Pb(II) removal.^13^^,^^14^ The sulfonation after hydroxylation is simple and feasible, and it is one of the most effective strategies to greatly improve the adsorption capacity and selective capture ability of Pb(II).^15^^,^^16^ Despite the large numbers of functional polymeric adsorbents contain -SO_3_H have been crafted, the successful large-scale applications are still limited by many challenges. Stacked and entangled of excessive more bulky -SO_3_H groups may result in large steric hindrance, affording insufficient reactions of some deep-buried active sites within the architectures will leading to worse ability to binding with Pb(II).^17^^,^^18^ With these considerations in mind, the integration of hydroxyl functional groups and sulfonic groups to co-modify the PAFs for forming bifunctional adsorbent provides a potential solution for promoting the site optimization and steric hindrance regulation.

In the present work, we reported a facile approach to prepare the -OH and -SO_3_H bifunctional SPAFs for Pb (II) selective removal from water. Series of adsorbents were prepared by modifying the pristine framework (PAF). The PAF functionalized by -OH to obtain the PAF-1-OH, increasing the number of -OH to gain the PAF-3-OH, and then SPAFs were prepared by introducing the different proportions of -SO_3_H groups to post-modified PAF-3-OH. Consequently, batch adsorption experiments were proceeded to test the adsorption performance of prepared adsorbents. SPAF-0.75 exhibits highest efficient adsorption performance for the capturing of Pb(II), and can achieve a maximum adsorption capacity of 212.34 mg g^−1^ for Pb(II) in aqueous solution. Structural characterization and theoretical calculations were combined in this study to discuss the adsorption mechanisms between Pb(II) and SPAF-0.75 in detail. Meanwhile, selectivity and reusability are also discussed. This work is the first to tailor the most suitable steric hindrance in PAFs for efficient selective capture of Pb(II) through systematically regulate the ratio of -OH and -SO_3_H.

## Results

### Characterizations of adsorbents

**Figure 1:**
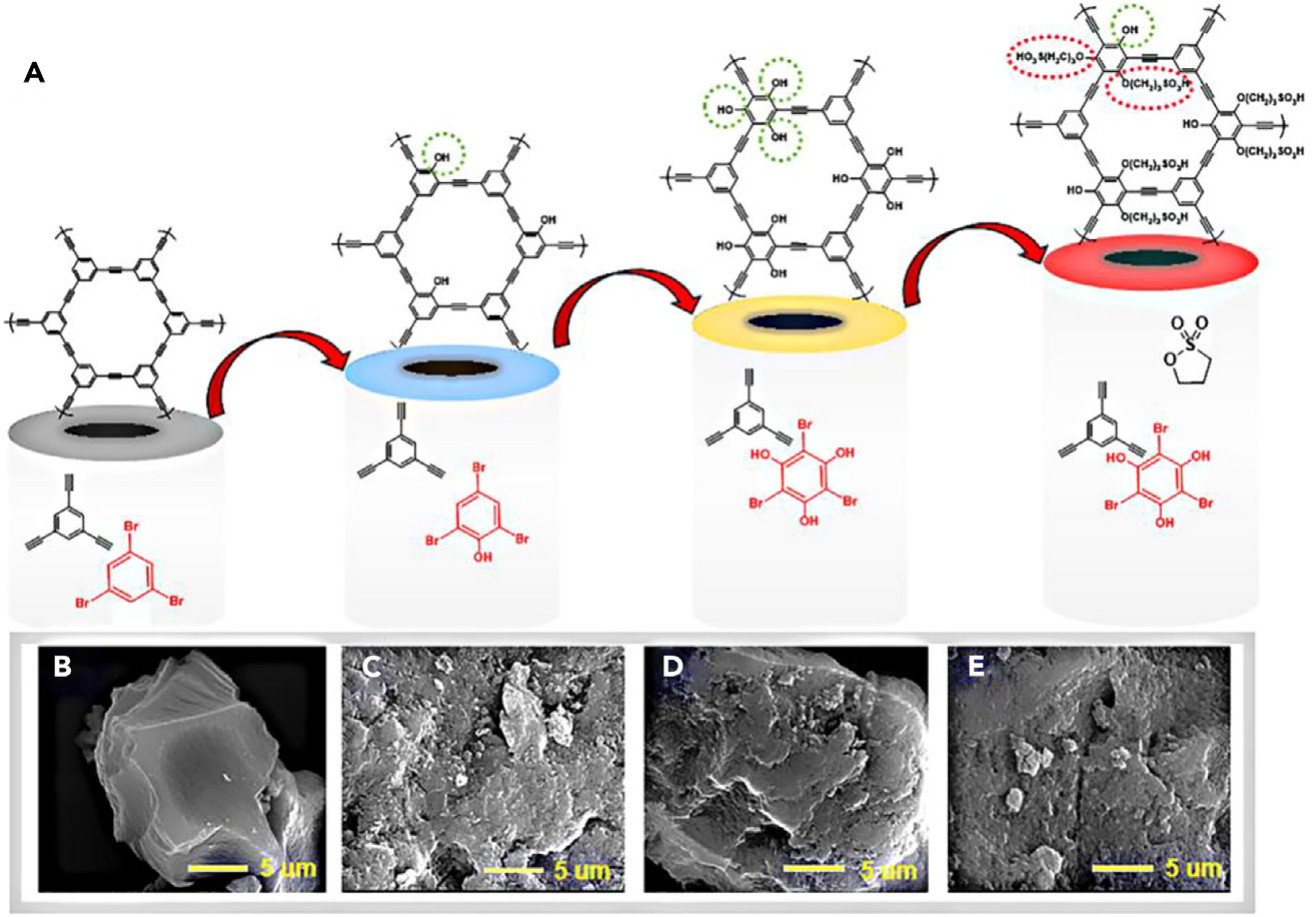
The schematic preparation and morphology explanation of PAFs (A) Procedure of preparing the PAF, PAF-1-OH, PAF-3-OH, SPAF-0.75. (B–E) SEM images of (B) PAF, (C) PAF-1-OH, (D) PAF-3-OH, (E) S-PAF-0.75.

To facilitate additional active sites, the introduction and optimization of -OH functional groups onto the pristine framework (PAF) was desired to prepare PAF-1-OH and PAF-3-OH. The presence of -OH groups allowed the covalent modification of quantitative -SO_3_H on the PAF-3-OH for preparing the SPAF-0.75 with the most suitable steric hindrance (Figure 1A). The morphologic features of the as-prepared adsorbents were performed by scanning electron microscopy (SEM), which clearly revealed the characteristics of their surface structure. As can be seen from Figure 1B, The surface of the pristine porous aromatic frame (PAF) is relatively flat and regular, PAF-1-OH (Figure 1C), PAF-3-OH (Figure 1D) and SPAF-0.75 (Figure 1E) functionalized by 1-OH, 3-OH and 0.75-SO3H groups showed the rough surface and the overall reflect irregular shape, and the expansion of the volume of the groups modifying PAF leads to an increase in its surface roughness.

**Figure 2:**
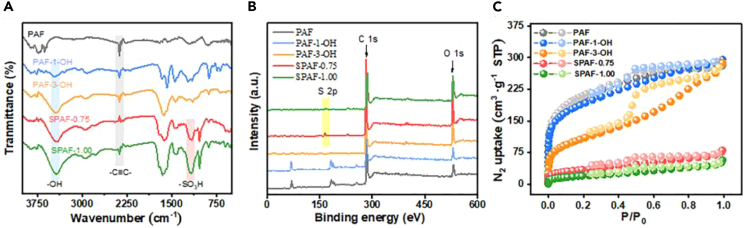
The systematic characterizations of prepared PAFs (A–C) FT-IR (A), XPS full scan survey spectra (B) and BET nitrogen gas adsorption-desorption isotherms (C) of PAF, PAF-1-OH, PAF-3-OH, SPAF-0.75 and SPAF-1.00.

The chemical structures of prepared adsorbents were characterized by FT-IR spectra to inspect the preparation process (Figure 2A). The absorption peaks of all prepared adsorbents at 2250 cm^−1^ are attributed to the stretching vibration of the –C≡C–, which indicated that the S-H cross-coupling reaction of all materials occurred successfully.^19^^,^^20^ For PAF-1-OH and PAF-3-OH, the stretching vibration of -OH groups at 3440 cm^−1^ could be observed, which indicated that -OH had been successfully modified onto the PAFs.^21^^,^^22^ The -OH peak areas of PAF-3-OH is larger than PAF-1-OH, indicating that the PAF-3-OH nanoparticles possess the higher hydroxyl densities. In addition, the characteristic peaks near 1172 cm^−1^ are stretching vibrations from -SO_3_H, and the gradual increase of -SO_3_H peak area of SPAF-0.25, SPAF-0.50, SPAF-0.75 and SPAF-1.00 further confirmed the introduction of different content of sulfonic group to PAF-3-OH (Figure S1A).^23^ The elemental composition of the obtained adsorbent can be further determined by XPS analysis, and the results are shown in Figure 2B, Figure S1B. The XPS full spectrum of all adsorbents confirmed the existence peak of O 1s and all SPAFs possess the peak of S 2p, indicating that the -OH and -SO_3_H was successfully introduced into the PAFs and PAF-3-OH, respectively.^24^ Moreover, the N_2_ adsorption-desorption experiments recorded at 77 K indicate PAF has a higher Brunauer-Emmett-Teller (BET) surface area of 736.83 m^2^ g^−1^ than PAF-1-OH (662.66 m^2^ g^−1^) PAF-3-OH (388.59 m^2^ g^−1^), SPAF-0.75 (122.36 m^2^ g^−1^) and SPAF-1.00 (70.71 m^2^ g^−1^), which account for the coupled functional groups clog the pore channels of PAF lead a trend of decreasing surface area (Figure 2C).^25^ The corresponding total pore volume and average pore diameter are presented in Table S1.

### Experimental combined theoretical assessment of adsorption performance

**Figure 3:**
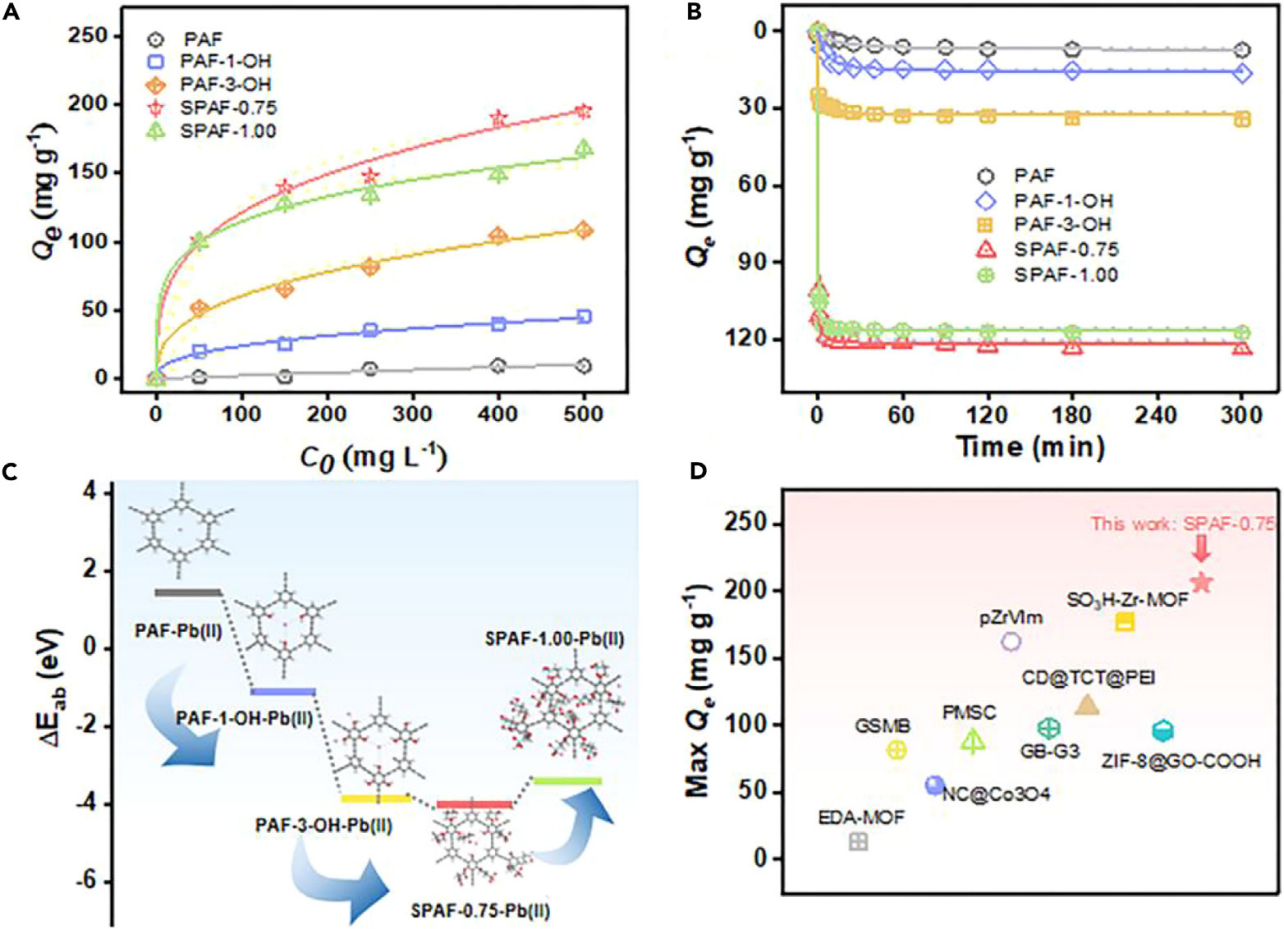
The combined experimental and theoretical evaluation of adsorption performance (A and B) (A) Adsorption isotherms and (B) adsorption kinetics for the adsorption of Pb(II) by PAF, PAF-1-OH, PAF-3-OH, SPAF-0.75 and SPAF-1.00. (C) The adsorption energy and adsorption models between prepared adsorbents and Pb(II). (D) Comparison of Pb(II) adsorption properties of different adsorbent.

Considering the features that Pb(II) forms metal hydroxide precipitates at alkaline conditions, the effect of pH on the Pb(II) adsorption capacity of prepared adsorbents were carried out in the pH range of 1–6.^26^ As observed in Figure S2A, the adsorption performance of prepared adsorbents for Pb(II) has been slightly increased with the increased pH values, reaching the maximum adsorption capacity at pH 6. The adsorption isotherms were carried out at the optimum condition of pH 6 and collected in Figure 3A. It can be seen that the SPAF-0.75 (212.34 mg g^−1^) is equipped with the best adsorption capacity of Pb(II) compared with PAF (9.3 mg g^−1^), PAF-1-OH (45.30 mg g^−1^), PAF-3-OH (108.50 mg g^−1^), SPAF-0.25 (142 mg g^−1^), SPAF-0.50 (146 mg g^−1^), SPAF-1.00 (168 mg g^−1^) (Figure S2D). The Langmuir model and the Freundlich model were chosen to describe the Pb(II) adsorption process (Figure S2C). The isotherm parameters are summarized in Table S2. A comparison of the coefficients of determination (R^2^) indicates that the Freundlich model describes the data slightly better than the Langmuir model, demonstrating that the multilayer adsorption caused by the asymmetrical adsorbent surface.^27^ The adsorption rate was evaluated in Figure 3B, SPAF-0.75 enabled rapid capture of aqueous Pb(II), and the highest adsorption equilibrium was reached within 10 min. In order to further understand the process of Pb(II) adsorption on prepared adsorbents, the data of was fitted by the pseudo-first-order and pseudo-second-order kinetic model (Figure S2B). The R^2^ value shows that the adsorption behavior was well described by the pseudo-second-order model (Table S3), indicating that chemisorption is the key step of rate-controlling in the capture of Pb(II).^28^ The full spectrum of XPS showed that SPAF-0.75 possess the strongest Pb 4f peaks compared with PAF-1-OH and PAF-3-OH after the adsorption of Pd(II) (Figure S3).^29^ The EDS of SPAF-Pb(II) as showed in Figure S4, the maximum distribution of Pb(II) was observed in SPAF-0.75-Pb(II), indicating the optimum introduction ratio of sulfonic acid group is 0.75.^30^ Furthermore, DFT calculations were performed to elucidate the relationship between the electronic properties of prepared adsorbent and the capture performance of Pb(II). As exhibited in Figure 3C, the *E*_ad_ values were determined to be 1.43, −1.03, −3.87, −3.41 and −3.99 eV for PAF-Pb(II), PAF-1-OH-Pb(II), PAF-3-OH-Pb(II), SPAF-1.00-Pb(II) and SPAF-0.75-Pb(II), respectively, indicating that SPAF-0.75 turned out to be the most efficient in Pb(II) removal theoretically.^31^ Finally, the Pb(II) adsorption capacities of the bifunctional SPAF-0.75 was compared with those of other adsorbents reported in recent years. The adsorption capacities of SPAF-0.75 for absorbing Pb(II) rank first (Figure 3D).^32^^,^^33^^,^^34^^,^^35^ Hence, the SPAF-0.75 exhibits a significantly higher Pb(II) adsorption capacity than the other adsorbents. This result has important implications for water treatment.

### 3.3 mechanism analysis for determining the binding sites of SPAF-0.75

**Figure 4:**
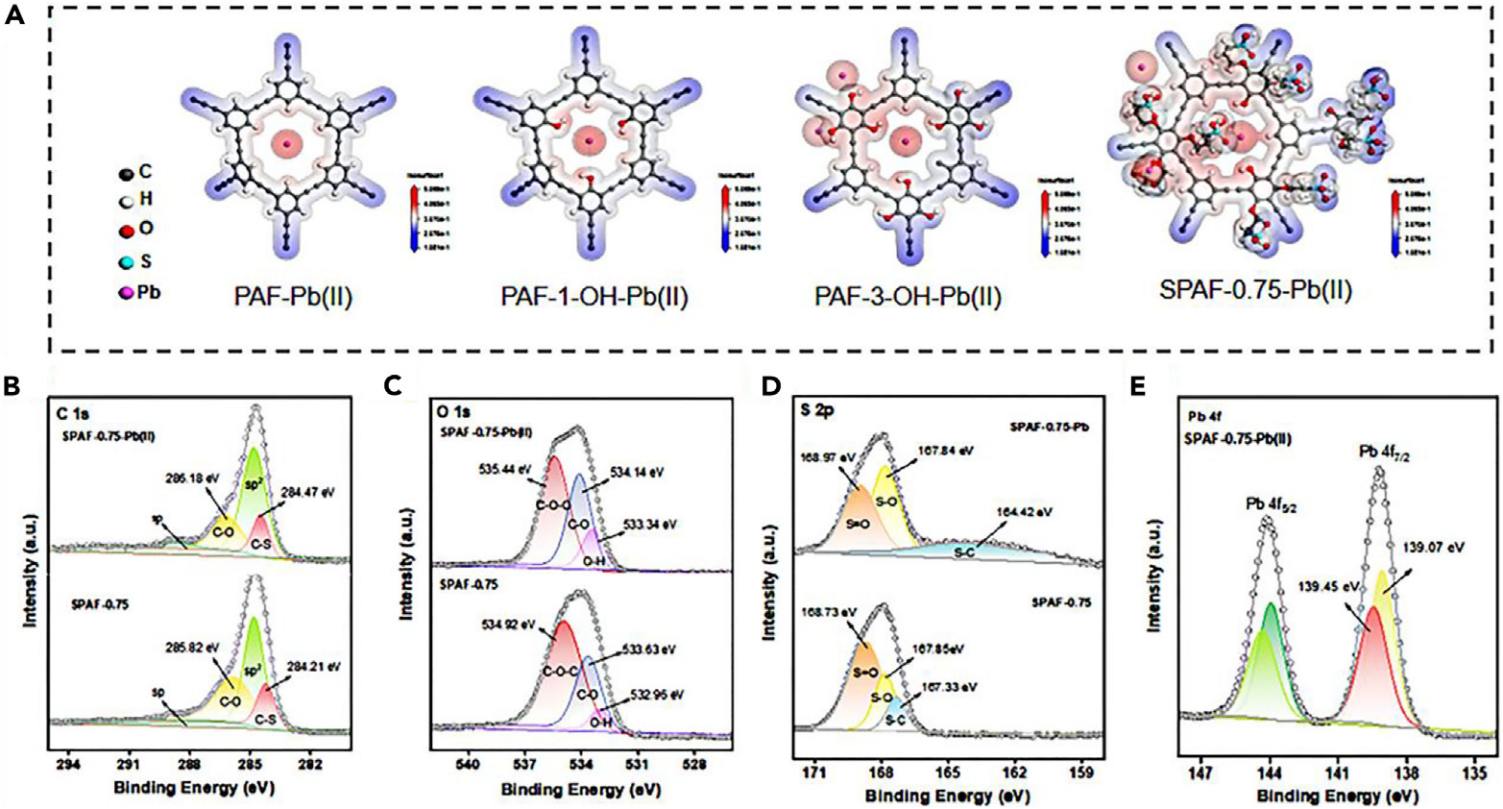
The mechanism analysis for determining the binding sites of PAFs (A) The surface electrostatic potential distribution (via DFT calculation) of PAF-Pb(II), PAF-1-OH-Pb(II), PAF-3-OH-Pb(II) and SPAF-0.75-Pb(II). (B–E) The blue to red color stands for more negative to positive potential, (B) C 1s fine XPS spectra (C),O 1s fine XPS (D) and S 1s fine XPS spectra before and after the SPAF-0.75 adsorption of Pb(II); (E) Pb 4f fine XPS spectra after the capturing of Pb(II).

To further clarify the adsorption mechanism of Pb(II) capture on prepared adsorbents, the atomic charges the distribution of the PAF-Pb(II), PAF-1-OH-Pb(II), PAF-3-OH-Pb(II) and SPAF-0.75-Pb(II) were performed by Mulliken population analysis of DFT calculations were presented in Figure 4A and Figures S5 and S6. For PAF, the all regions possessed the positive electrostatic potential, indicating that PAF cannot provide any sites for capturing Pb(II). For PAF-1-OH and PAF-3-OH, the regions of O possessed a more negative electrostatic potential than other regions, indicating -OH groups as adsorption sites to coordinate with Pb(II). For SPAF-0.75, the adjacent regions of -OH and -SO_3_H all exhibited the negative potential tend to combine the Pb(II) with positive charge.^36^ In order to further investigate the major contributing functional groups and electronic properties of SPAF-0.75, XPS characterization was conducted on the adsorbent before and after metallization. As illustrated in Figures 4B and 4C, The C 1s spectra with characteristic peaks at around 285.82 and 284.47 eV corresponded to the C-O, C-S, and the peaks of O 1s spectra at 534.92、533.63、532.96 eV were attributed to C-*O*-C, C-O, O-H. After Pb(II) ions adsorbed, the energy peaks of C-O and O-H transferred to the higher binding energy, demonstrating the -OH groups participated in the adsorption process.^37^^,^^38^ Meanwhile, the S 2p spectra of SPAF-0.75 showed three peaks at 168.73, 167.85 and 167.33 eV assigned to S=O, S-O and S-C bonds, respectively (Figure 4D). The peaks of S=O shifted toward higher binding energy after capturing Pb(II). It revealed that the -SO_3_H groups appeared in different degrees of electron consumption in binding with Pb(II).^39^ At the same time, two peaks at 139.21 eV and 144.11 eV of the Pb 4f spectra were attributed to the binding energy of Pb 4f_7/2_ and Pb 4f_5/2_, respectively, in Figure 4E.^40^ The distance of binding energy between the two peaks is 4.90 eV, which is close to the standard value of 4.87 eV, indicating that the Pb element adsorbed on the surface of SPAF-0.75 is mainly in the form of Pb(II). Additionally, Pb-O (139.07 eV) and Pb-OSO_2_ (139.45 eV) peaks appeared in the Pb 4f_7/2_ XPS spectrum of SPAF-0.75-Pb(II).^41^ The results of XPS analysis strongly supported the fact that -OH and -SO_3_H jointly provide the chemical interactions to drive the Pb(II) adsorption onto SPAF-0.75 surface.

### Adsorption selectivity and practical application of SPAF-0.75

**Figure 5:**
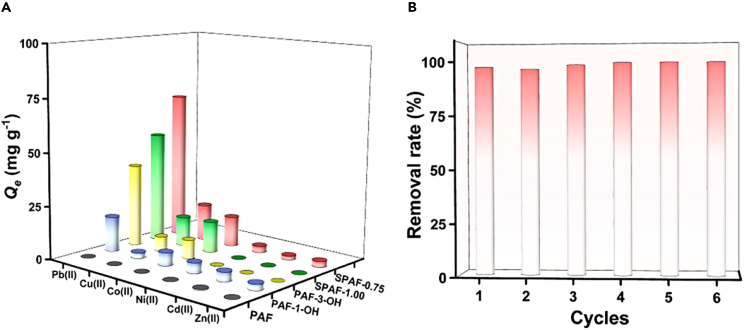
The assessment of the application potentials in selective capture of Pb(II) (A) Selective adsorption of Pb(II) by five prepared adsorbents in the mixture of multiple interfering ions. (B) Regeneration and recyclability of the SPAF-0.75.

In view of the special functions of Pb(II), it is particularly important to evaluate the ability of adsorbents for selective capture of Pb(II). Multiple metal ions including Cu(II), Co(II), Ni(II), Cd(II), and Zn(II) were used to form the competitive adsorption environment for Pb(II). The selective results of prepared adsorbents were shown in Figure 5A, SPAF-0.75 has the better adsorption capacity for Pb(II) with the highest removal rate than other materials. As shown in the Figure S7, the *β* values of Pb^2+^/Cu^2+^, Pb^2+^/Co^2+^, Pb^2+^/Ni^2+^, Pb^2+^/Cd^2+^ and Pb^2+^/Zn^2+^ were 2.13, 11.27, 12.40, 24.80 and 31.0, respectively, revealing prominent adsorptive selectivity of SPAF-0.75 toward Pb(II) in coexistence systems of metal cations. Meanwhile, the adsorption properties of SPAF-0.75 were also greatly improved compared with PAF-3-OH and pristine PAF. These results proved that SPAF-0.75 is equipped with superior selectivity for the capturing of Pb(II) in the water environment with the coexistence of multi-ions. The reusability performance of an adsorbent is significant for practical engineering applications. The reusability of SPAF-0.75 was evaluated by cyclic regeneration with thiourea and HNO_3_ mixed solution as the eluent. The Pb(II) recovery efficiency still retain 100% after six adsorption-desorption cycles (Figure 5B), indicating that SPAF-0.75 can be utilized for the sustainable decontamination of Pb(II). All these results indicated that SPAF-0.75 provided great application potentials in the capture of Pb(II) from wastewater.

## Discussion

In summary, starting from sterically hindered building blocks, we have been able to construct PAFs with target topology by adjusting steric hindrance effects. Through rational design, we successfully synthesized a bifunctional SPAF-0.75 by regulating the contents of the introduction of -SO_3_H to post-modifiy the PAF-3-OH with the sites optimization. As a result, SPAF-0.75 affords a significant improvement in the binding of Pb(II) ions on the functionalized aromatic rings. The obtained SPAF-0.75 adsorbent features a desirable removal performance, and remarkable adsorption selectivity in the capture of aqueous Pb(II), achieving a maximum adsorption capacity of 212.34 mg g^−1^. Furthermore, the removal rate of SPAF-0.75 adsorption Pb(II) still maintain at 100% after six regeneration cycles. Systematical characterizations and DFT calculations certified the -OH and -SO_3_H groups as co-binding sites to capture of Pb(II), and elucidated that the satisfactory selectivity of Pb(II) was owing to the most suitable spatial configuration formed by coordinating with SPAF-0.75. In addition to the obtained high performance, this approach also gives a rise to extend the understanding of structure-property relationship. We anticipate this study will certainly provide a promising strategy to construct targeted functional PAFs for diverse applications in the future.

### Limitations of the study

It is undoubtedly that the development and innovation of the cost-effective adsorbents is the main keynote of the future research direction. In the future, this type of research is also required a further study in regard to the future applications of actual Pb(II)-wastewater treatment.

## STAR★Methods

### Key resources table

**Table:** 

REAGENT or RESOURCE	SOURCE	IDENTIFIER
**Chemicals, peptides, and recombinant proteins**		

1, 3, 5-Triethynylbenzene	J&K Scientific Co., Ltd	CAS: 7567-63-7
1, 3, 5-tribromobenzene	J&K Scientific Co., Ltd	CAS: 626-39-1
2, 4, 6-tribromophenol	J&K Scientific Co., Ltd	CAS: 118-79-6
Triethylamine	J&K Scientific Co., Ltd	CAS: 121-44-8
N, N-dimethylformamide	J&K Scientific Co., Ltd	CAS: 68-12-2
Tetrakis (triphenylphosphine) palladium (Pd(0))	J&K Scientific Co., Ltd	CAS: 14221-01-3
Cuprous iodide	J&K Scientific Co., Ltd	CAS: 7681-65-4
2, 4, 6-tribromo-phenyl-1.3.5-triol	Bide Pharmatech Ltd.	CAS: 3354-82-3
1, 3-propyl sulfonolactone	Aladdin Co., Ltd.	CAS: 1120-71-4
Isopropyl alcohol	Aladdin Co., Ltd.	CAS: 57-55-6
Leadr nitrate (PbNO_3_)	Aladdin Co., Ltd.	CAS: 10099-74-8
Sodium hydroxide	Xilong Chemical Co.,Ltd.	CAS: 1310-73-2

### Resource availability

#### Lead contact

Further information and requests for resources and reagents should be directed to and will be fulfilled by the lead contact, Xubiao Luo (luoxubiao@126.com) or Hui Shi (shihui900501@126.com).

#### Materials availability

This study did not generate new unique reagents.

### Experimental model and study participant details

Our study does not use any experimental models.

### Method details

#### The method of synthesis of adsorbent

##### The synthesis of PAFs

Triethylamine (6.7 mL), DMF (6.7 mL) a magneton were added into a round-bottom flask (50 mL) in sequence. And then, magnetic stirring was carried out at room temperature, and argon degassing was carried out for 15 min; 1, 3, 5-triacetylene benzene (150.18 mg), 1, 3, 5-tribromobenzene (330 mg), tetraphenylphosphadium (0) (65 mg), cuprous iodide (15 mg) were added to the above round-bottomed flask in turn, and magnetic agitation was continued at room temperature, while argon degassing was performed for 20 min; After the reaction system is vacuumed, it is placed in a constant temperature magnetic oil bath, and the temperature is set at 80°C. Then the reaction reflux is carried out at this temperature for 72 h (the whole reaction process should be carried out under the condition of avoiding light); After the reaction is over, the obtained polymer is soaked in trichloromethane for 4–5 h, and then pumped and filtered with trichloromethane, water, acetone and methanol in order to wash away some unreacted monomers and impurities. Finally, the product is dried under vacuum at 65°C for 24 h to obtain the basic porous aromatic skeleton, which is named PAF.

#### The preparation of PAF-1-OH and PAF-3-OH

PAF-1-OH was synthesized by Sonogashira cross-coupling reaction catalyzed by palladium. 1, 3, 5-tribromophenyl-2-ol (350 mg), 1, 3, 5-triacetylidene benzene (150.18 mg), tetri (triphenylphosphine) palladium (0) (65 mg) and cuprous iodide (15 mg) were dissolved in DMF and triethylamine mixture (6.7 mL/6.7 mL), and after several degasses treatment, The reaction temperature was set at 105°C, and the reaction mixture was reflux in N_2_ atmosphere for 72 h. At the end of the reaction, trichloromethane, ethanol and acetone were extracted and filtered in order to remove the unreacted guest solvent and catalyst. Further purification of the polymer was performed by methanol Soxhlet extraction for 48 h. The modified material PAF-1-OH was prepared by vacuum drying at 80°C for 24 h.

1, 3, 5-tribromo-phenyl-2-ol was replaced with 2, 4, 6-tribromo-phenyl-1, 3, 5-triol, other steps remained unchanged, and the number of grafted -OH functional groups was increased to obtain the material PAF-3-OH.

#### PAF-3-OH was modified by -SO_3_H to obtain a series of SPAF adsorbents

The prepared PAF-3-OH (241.268 mg, 1 mmol), sodium hydroxide (0.12 g, 1 mmol), isopropyl alcohol (20 mL) and distilled water (1 mL) were stirred at 80°C for half an hour. Then, 1, 3-propyl sulfonolactone (200 μL) was added to the reaction solution and heated at the same temperature for 16 h. At the end of the reaction, the PAF-SO_3_Na product is obtained, which is cooled to room temperature and eluted with trichloromethane, ethanol and acetone for several times to remove the unreacted reagent. Completely sulfonated SPAF was obtained by ion exchange for 12 h using 1 M sulfuric acid solution as solvent. Further purification of the polymer was performed by methanol Soxhlet extraction for 48 h. The modified material SPAF-1.00 was prepared by vacuum drying at 80°C for 24 h. By changing the amount of 1, 3-propyl sulfonolactone added, SPAF-0.25, SPAF-0.50 and SPAF-0.75 were obtained respectively.

#### Batch experiments

##### Isothermal adsorption

The adsorption capacity was calculated using Equation 1.

(Equation 1) $$Q_{e}=\frac{\left(C_{0}-C_{e}\right)v}{m}$$

Where *Q*_e_ is the adsorptions capacity (mg g^−1^), *C*_0_ and *C*_e_ denotes the initial and equilibrium concentrations of metals ions (mg L^−1^), respectively, v represents the volume of the metal ion solution (L), and m is the adsorbent mass (g).

Langmuir (Equation 2) and Freundlich (Equation 3):

(Equation 2) $$\frac{C_{e}}{Q_{e}}=\frac{C_{e}}{Q_{m}}+\frac{1}{K_{L}Q_{m}}$$

(Equation 3) $$\log\;Q_{e}=\frac{{\log\;C}_{e}}{n}+\log\;k_{f}$$

Where *Q*_e_ is the mass of adsorbed at equilibrium (mg g^−1^); and *C*_e_ is the equilibrium concentration (mg L^−1^) in the solution. In Equation 4, *Q*_m_ (mg g^−1^) and K_L_ (L mg^−1^) are the Langmuir parameters; *Q*_m_ represents the maximum adsorption capacity; and K_L_ is the adsorption equilibrium constant. In Equation 5, K_f_ (mg^1−(1/n)^L^1/n^g^−1^) and n are the Freundlich parameters.

##### Adsorption kinetics

The pseudo-first-order and the linearized pseudo-second-order models are as follows:

(Equation 4) $$\ln\left(Q_{e}-Q_{t}\right)=-k_{1t}+\ln\;Q_{e}$$

(Equation 5) $$\frac{t}{Q_{t}}=\frac{t}{Q_{t}}+\frac{1}{h_{0}}$$

(Equation 6) $$h_{0}=k_{2}Q_{e}^{2}$$

Where *Q*_e_ represent the mass of adsorbent adsorbed at equilibrium (mg g^−1^); *Q*_t_ denotes the mass of adsorbent adsorbed (mg g^−1^) at time t (min); and K_1_ (min^−1^), K_2_ (g mg^−1^ min^−1^) and *h*_0_ (mg g^−1^ min^−1^) are the rate constants for the pseudo-first-and pseudo-second-order adsorption models, respectively.

#### Adsorption experiment

The effect of pH on the adsorption performance of prepared adsorbents were investigated by adjusting the pH 1–6 of the Pb(II) solution (50 mg L^−1^) by HNO_3_. The isotherm adsorption experiment of Pb(II) was carried out by adding 10 mg prepared adsorbents into 20 mL Pb(II) solution with different concentrations in Erlenmeyer flask and shaken at 25°C for 24 h. The kinetic adsorption was performed by a system containing 100 mg prepared adsorbents and 200 mL Pb(II) solution (100 mg L^−1^) within 300 min. The selectivity of prepared adsorbents capture of Pb(II) was performed in the mixture of co-existing metal ions of Pb(II), Cd(II), Cu(II), Zn(II), Ni(II), Co(II), and the initial concentration of all metal ions is 2 mmol L^−1^. And then, the mixed solution follow the rule of 10 mg prepared adsorbents and 20 mL mixture in sealed conical flasks, and shaken for 12 h under 25°C. Six adsorption-desorption cycles were carried out to judge the recycling availability of SPAF-0.75. The Pb(II) adsorbed on SPAF-0.75 was eluted with 1M HNO_3_ and 1M thiourea, and the regenerated adsorbent was dried at 65°C for 12 h and then used in the next cycle of adsorption experiment.

#### Characterizations

The morphology of the synthesized samples was characterized by scanning electron microscope (SEM, FEI, America). Fourier transmission infrared spectra (FT-IR, 4000-400 cm^−1^) were logged recorded on a NICOLET NEXUS 4700 FT-IR spectrometer (Nicolet, Tacoma Washington, USA). X-ray photoelectron spectroscopy (XPS) was recorded by a Kratos XSAM800 spectrometer utilizing Al target (1486.6 eV) X-ray source (Kratos, UK). The Brunauer-Emmett-Teller (BET) surface area measurement was performed by N_2_ adsorption-desorption isotherms at the liquid nitrogen temperature (77 K) using Micromeritics TriStar II 3020. The metal ions concentrations was measured using A ConteAA 700 (Analytik Jena, Germany) flame atom absorption spectrometer (AAS).

#### Theoretical calculations

Density functional theory (DFT) calculations were performed using the Materials Studio Dmol3 program package to explore electrostatic potential distribution of prepared adsorbents and the adsorption energy of binding with Pb(II). By considering the exchange correlation functional, the spin-unrestricted polarization DFT on the basis of generalized gradient approximation (GGA) with the correlation energy function of Becke–Lee–Yang–Parr (BLYP) was used to optimize the molecule geometrically. The double numerical plus polarization (DNP) basis set was used, and the geometric optimization was carried out to obtain the most stable adsorption structure with the minimum total energy.

## Data Availability

Any additional information required to reanalyze the data reported in this paper is available from the lead contact upon request.

## Supplemental information

Document S1. Figures S1‒S7 and Tables S1‒S3
