# Supplementary material for: Regulating steric hindrance in difunctionalized porous aromatic frameworks for the selective separation of Pb(II)
Source: iScience. 2023 Oct 19;26(11):108274. doi: 10.1016/j.isci.2023.108274 (PMC10665823; doi:10.1016/j.isci.2023.108274)
Supplement: Document S1. Figures S1‒S7 and Tables S1‒S3 [file mmc1.pdf]

iScience, Volume *26*

Supplemental information

Regulating steric hindrance in difunctionalized porous aromatic frameworks for the selective separation of Pb(II)

Xuan Ding, Jiayi Liu, Hui Shi, Zhou Yi, Lei Zhou, Wei Ren, Penghui Shao, Liming Yang, Derun Zhao, Yun Wei, and Xubiao Luo

Supporting Information

Regulating steric hindrance in difunctionalized PAFs for selective separation of Pb(II)

Xuan Ding[a], Jiayi Liu[a], Hui Shi[a,*], Zhou Yi[c], Lei Zhou[a], Wei Ren[a], Penghui Shao[a], Liming Yang[a], Derun Zhao[a], Yun Wei[a], Xubiao Luo[a,b,*]

Supplementary Figures and Tables

Figure S1.

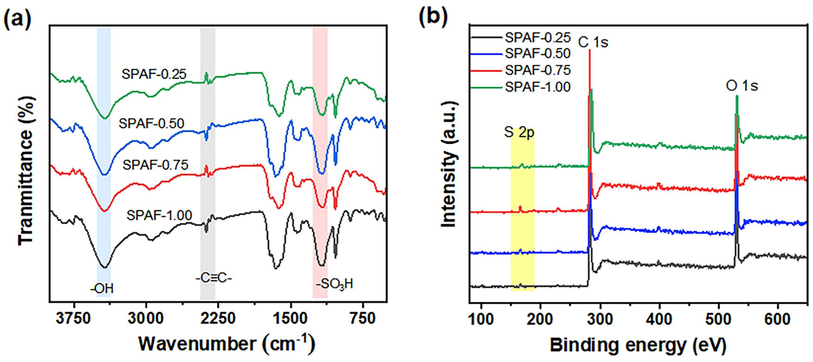

Supplementary Figure1. The characteristics further confirmed the introduction of different content of sulfonic group to PAF-3-OH, related to Figure 2

(a) FT-IR of SPAF-0.25, SPAF-0.50, SPAF-0.75 and SPAF-1.00.

(b) XPS full scan survey spectra of SPAF-0.25, SPAF-0.50, SPAF-0.75 and SPAF-1.00.

Figure S2.

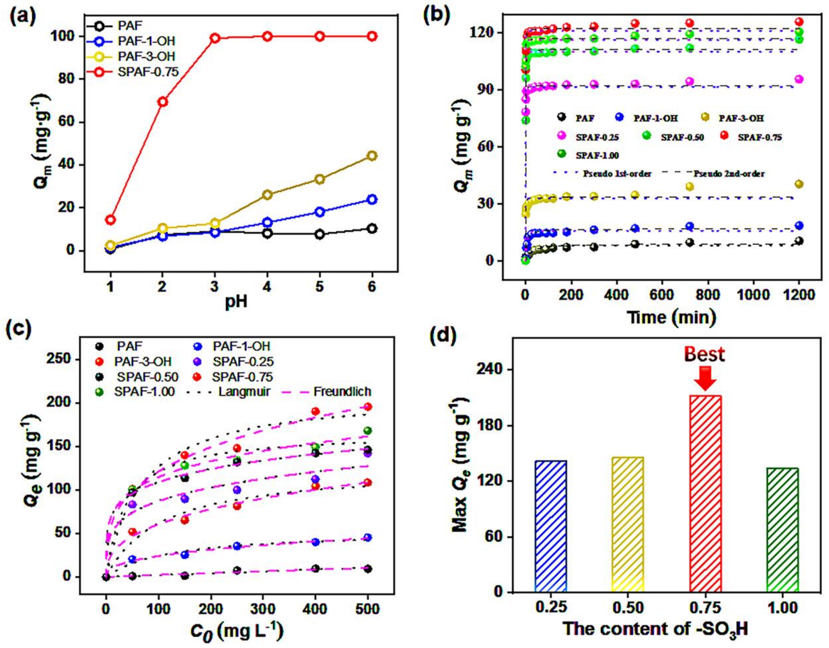

Supplementary Figure2. Batch adsorption experiments of all prepared PAFs, related to Figure 3

(a) The effect of pH on the adsorption of Pb(II) by PAF, PAF-1-OH, PAF-3-OH and SPAF-0.75.

(b) adsorption kinetics and Adsorption isotherms (c) for Pb(II) on all prepared adsorbents.

(d) Comparison of Pb(II) adsorption properties of different content of -SO_3H

Figure S3.

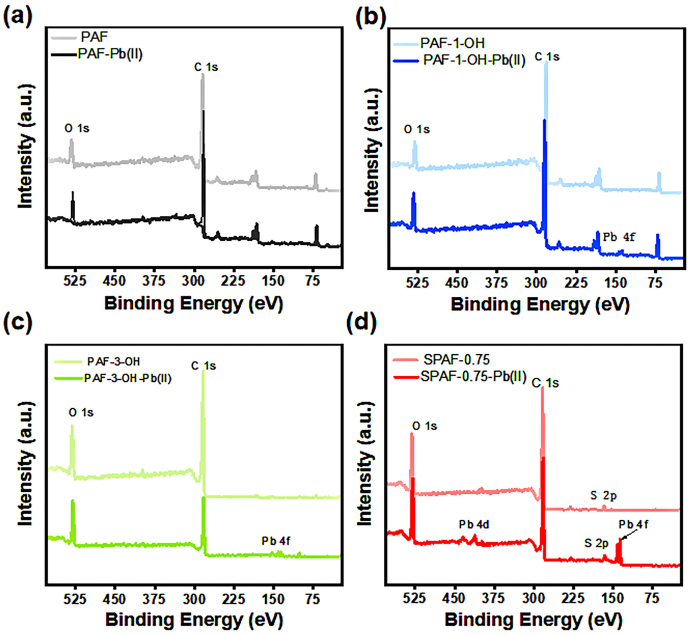

Supplementary Figure3. The full spectrum of XPS of prepared PAFs before and after adsorption of Pd(II), related to Figure 3

(a) PAF.

(b) PAF-1-OH.

(c) PAF-3-OH.

(d) SPAF-0.75.

Figure S4.

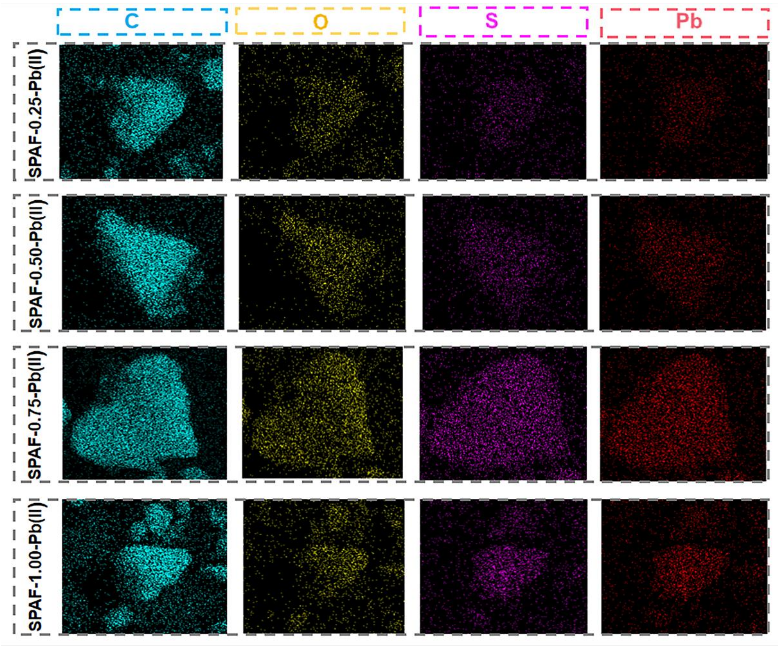

Supplementary Figure4. The Mapping images of prepared SPAFs after adsorption of Pd(II), related to Figure 3

Mapping images of SPAF-0.25-Pb(II), SPAF-0.50-Pb(II), SPAF-0.75-Pb(II), SPAF-1.00-Pb(II) and SPAF-0.25-Pb(II).

Figure S5.

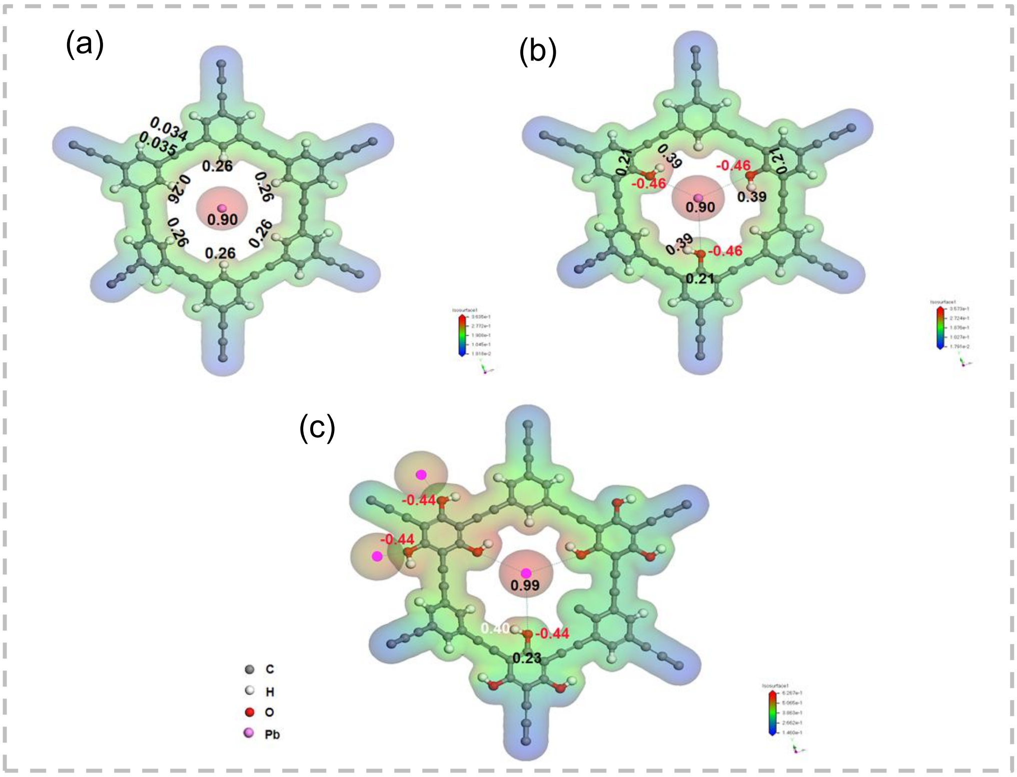

Supplementary Figure5. The detail of the surface electrostatic potential distribution (via DFT calculation), related to Figure 4(a)

(a) PAF-Pb(II).

(b) PAF-1-OH-Pb(II).

(c) PAF-3-OH-Pb(II).

Figure S6.

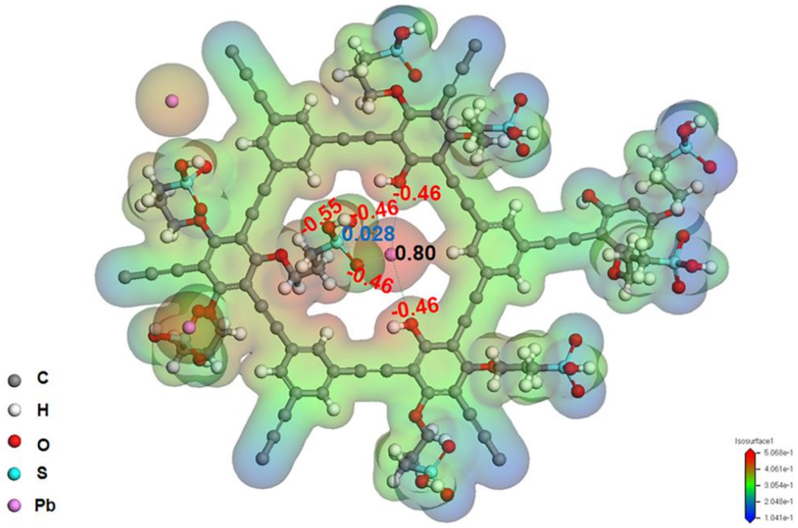

Supplementary Figure6. The detail of the surface electrostatic potential distribution (via DFT calculation) of SPAF-0.75-Pb(II), related to Figure 4(a)

Figure S7.

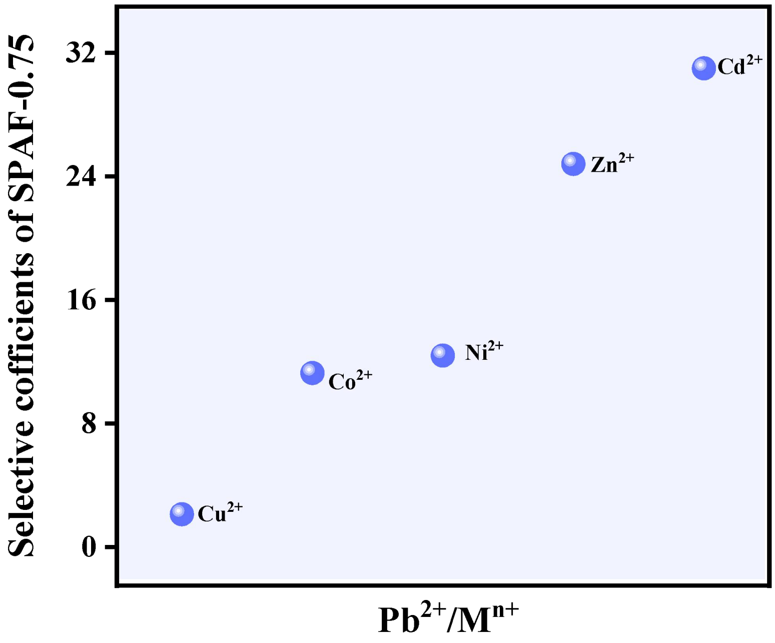

Supplementary Figure7. Selectivity coefficient of SPAF-0.75 for Pb2+ removal in competitive systems of metal cations, related to Figure 5(a)

Adsorbents	BET （$m^2\ g^{-1}$）	Total pore volume （$m^3\ g^{-1}$）	Average pore diameter （nm）
PAF	736.83	0.4429	2.1555
PAF-1-OH	662.66	0.4568	2.1988
PAF-3-OH	388.59	0.4416	3.5233
SPAF-0.75	122.36	0.1222	3.2316
SPAF-1.00	70.71	0.0849	4.804

Table S1 Porosity parameters of the prepared adsorbents, related to Figure 2(c)

Table S2 Langmuir and Freundlich parameter values for the adsorption of Pb(II) on the prepared adsorbents at 298 K, related to Figure 3(a)

Adsorbent	Langmuir isotherm parameters			Freundlich isotherm parameters		
	Q_m (mg g^{-1})	KL (L mg^{-1})	R^2	n	KF ($mg^{1-(1/n)}L^{1/n}g^{-1}$)	R^2
PAF	10.68	3.65E-9	0.795	1.100	0.037	0.865
PAF-1-OH	52.57	8.64E-3	0.854	2.637	4.21	0.987
PAF-3-OH	125.94	9.60E-3	0.839	2.755	11.39	0.989
SPAF-0.25	142.34	2.59E-2	0.561	4.246	29.49	0.947
SPAF-0.50	146.89	2.67E-2	0.867	4.721	43.30	0.998
SPAF-0.75	212.05	3.14E-2	0.815	5.367	46.21	0.993
SPAF-1.00	166.10	1.48E-2	0.851	3.368	28.26	0.991

Table S3. Parameter values for the adsorption of Pb(II) on the PAFs at 298 K according to the pseudo-first-order and pseudo-second-order models, related to Figure 3(b)

Adsorbents	Pseudo-1st-order		Pseudo-2nd-order	
	Q_{e1} (mg g-1)	R_1^2	Q_{e2} (mg g-1)	R_2^2
PAF	6.54	0.862	7.20	0.902
PAF-1-OH	14.83	0.985	15.93	0.982
PAF-3-OH	31.38	0.952	31.95	0.973
SPAF-0.25	90.69	0.967	92.47	0.997
SPAF-0.50	107.05	0.877	107.05	0.877
SPAF-0.75	120.03	0.952	121.44	0.997
SPAF-1.00	115.38	0.993	116.44	0.999
